# Pyruvate kinase M2 regulates glucose metabolism by functioning as a coactivator for hypoxia-inducible factor 1 in cancer cells

**DOI:** 10.18632/oncotarget.299

**Published:** 2011-06-25

**Authors:** Weibo Luo, Gregg L. Semenza

**Affiliations:** ^1^Vascular Program, Institute for Cell Engineering, The Johns Hopkins University School of Medicine, Baltimore, USA; ^2^McKusick-Nathans Institute of Genetic Medicine, The Johns Hopkins University School of Medicine, Baltimore, USA; ^3^Department of Biological Chemistry, The Johns Hopkins University School of Medicine, Baltimore, USA; ^4^Department of Oncology, The Johns Hopkins University School of Medicine, Baltimore, USA; ^5^Department of Pediatrics, The Johns Hopkins University School of Medicine, Baltimore, USA; ^6^Department of Medicine, The Johns Hopkins University School of Medicine, Baltimore, USA; ^7^Department of Radiation Oncology, The Johns Hopkins University School of Medicine, Baltimore, USA

**Keywords:** HIF-1, hypoxia, metabolism, glycolysis, Warburg effect

## Abstract

Cancer cells feature altered glucose metabolism that allows their rapid growth. They consume large amounts of glucose to produce lactate, even in the presence of ample oxygen, which is known as the Warburg effect. Pyruvate kinase M2 (PKM2) contributes to the Warburg effect by previously unknown mechanisms. Hypoxia-inducible factor 1 (HIF-1) mediates *PKM2* gene transcription and metabolic reprogramming in cancer cells. The recent discovery of novel physical and functional interactions between PKM2 and HIF-1 in cancer cells has provided insight into molecular mechanisms underlying the Warburg effect.

## INTRODUCTION

Altered glucose metabolism is a key feature that distinguishes cancer cells from normal cells. Most cancer cells consume higher amounts of glucose and subsequently produce much more lactate than normal cells, even in the presence of ample O_2_. This phenomenon is known as the Warburg effect [1]. Since Otto Warburg made this important observation in 1924 [2], many researchers have attempted to elucidate the underlying molecular mechanisms in cancer cells. Hypoxia-inducible factor 1 (HIF-1), Myc, p53, Ras, Akt, Src, pyruvate kinase (PK) M2, and lactate dehydrogenase A (LDHA) have been implicated in the Warburg effect [3-8]. We recently discovered that the glycolytic enzyme PKM2 promotes the Warburg effect by serving as a transcriptional coactivator for HIF-1 in cancer cells [9]. This research perspective will discuss these recent findings regarding physical and functional interactions of HIF-1 and PKM2.

## HIF-1 AND METABOLIC REPROGRAMMING IN CANCER CELLS

HIF-1 is a heterodimeric transcription factor, consisting of an O_2_-regulated HIF-1α subunit and a constitutively expressed HIF-1β subunit [10, 11]. HIF-1 is a master regulator of transcriptional responses to reduced O_2_ availability (hypoxia), which is found in the majority of advanced human cancers [12, 13]. In well-oxygenated human cells, HIF-1α is hydroxylated at proline-402 and/or proline-564 by the prolyl hydroxylase domain proteins, PHD1-3 [14]. PHD2 is primarily responsible for regulating basal HIF-1α levels in cancer cells [15]. Prolyl-hydroxylated HIF-1α is bound by the von Hippel-Lindau (VHL) tumor suppressor protein, which is the substrate recognition component of an E3 ubiquitin-ligase complex, leading to HIF-1α protein degradation by the 26*S* proteasome [16]. Under hypoxic conditions, HIF-1α prolyl hydroxylation is inhibited, thereby stabilizing HIF-1α protein [17]. HIF-1α protein levels are also increased in normoxic cancer cells with loss of function of certain tumor suppressors, most notably VHL in the clear cell type of renal cell carcinoma [16, 18, 19]. HIF-2α is a paralog of HIF-1α that is also O_2_-regulated, dimerizes with HIF-1β, and transactivates a group of target genes that partially overlaps the battery of genes regulated by HIF-1 [20, 21].

**Figure 1:**
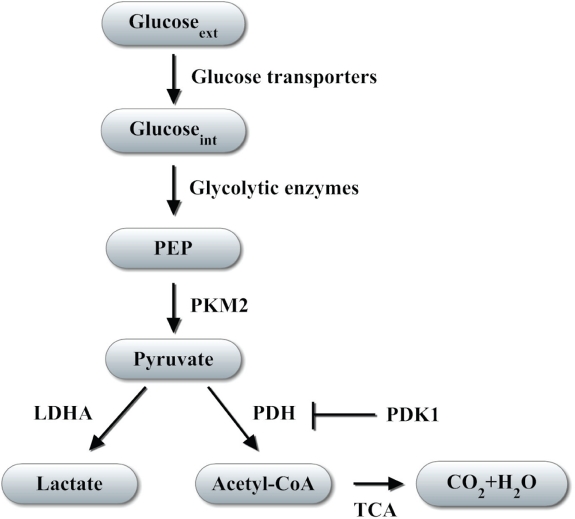
Regulation of glucose metabolism by HIF-1 HIF-1 controls transcription of genes encoding glucose transporters, which transport glucose from the extracellular (ext) to the intracellular (int) milieu, and glycolytic enzymes, which convert glucose to lactate as glycolytic end-product or acetyl coenzyme A (Acetyl-CoA) that is metabolized in the tricarboxylic acid cycle (TCA). Pyruvate kinase M2 converts phosphoenolpyruvate (PEP) into pyruvate, which is upstream of the decision point for glycolytic vs oxidative metabolism. Arrow indicates direction of glucose metabolism; blocked line indicates inhibition.

Activation of HIF-1 commonly occurs in many cancer types and is a driving force regulating many steps in cancer progression [18, 22]. HIF-1 activates the transcription of genes encoding proteins that mediate angiogenesis, invasion, metastasis, and the shift from oxidative to glycolytic metabolism [12, 18, 19, 22, 23]. By activating the transcription of genes encoding glucose transporters and glycolytic enzymes, HIF-1 enhances glucose uptake and glycolysis in cells [23-27]. HIF-1 also controls expression of LDHA and pyruvate dehydrogenase kinase 1 (PDK1) [25, 26, 28, 29]. LDHA catalyzes the conversion of pyruvate to lactate (Figure 1), thereby decreasing mitochondrial utilization of pyruvate as a substrate for pyruvate dehydrogenase (PDH), which converts pyruvate to acetyl coenzyme A (AcCoA). PDK1 phosphorylates the catalytic subunit of PDH, leading to its inactivation, which shunts pyruvate away from the mitochondria. HIF-1 activation shifts the balance of metabolism from oxidative phosphorylation toward glycolysis and mediates the Warburg effect in VHL-null renal carcinoma cells [30].

## REGULATION OF PKM2 EXPRESSION IN CANCER CELLS

PK catalyzes the conversion of phospho*enol*pyruvate to pyruvate (Figure 1) and is composed of M1-/M2-type and L-/R-type isoforms, which are encoded by *PKM2* and *PKLR* genes, respectively [31]. Tissue-specific promoters control expression of PKL, which is expressed in liver and kidney, and PKR, which is expressed in erythrocytes. PKM1 and PKM2 are the alternatively spliced products of the *PKM2* primary** RNA transcript with PKM1 and PKM2 mRNA containing sequences encoded by exon 9 or exon 10, respectively [32]. PKM1 is expressed in muscle and brain, whereas PKM2 is expressed in the embryo and in cancer cells. The transcription factors Sp1 and Sp3 bind to a GC-rich element in the promoter of the human *PKM2* gene [33]. Sp1 constitutively activates transcription of *PKM2*, whereas Sp3 functions as a transcriptional repressor that dissociates from the *PKM2* gene under hypoxic conditions.

We recently identified a hypoxia response element (HRE) within the first intron of the human *PKM2* gene [9]. Heterodimer complexes of HIF-1β with HIF-1α, but not HIF-2α, bound to the *PKM2* HRE and increased the activity of a luciferase reporter gene driven by the *PKM2* HRE in hypoxic HeLa cells. Mutation of the HIF-1 binding site in the *PKM2* HRE or knockdown of HIF-1α protein expression suppressed reporter gene activity. Hypoxia induced the expression of PKM1 and PKM2 mRNA in wild-type, but not in HIF-1α-knockout, mouse embryo fibroblasts [9].

PKM2 expression in cancer cells is also regulated by microRNAs (miRs). miR-326 matches two regions in the 3’-untranslated region (UTR) of PKM2 mRNA and transfection of miR-326 precursor decreased PKM2 3’-UTR-luciferase reporter activity and PKM2 protein levels in glioma cells [34]. miR-133a and miR-133b are also implicated in PKM2 expression. PKM2 overexpression is associated with downregulation of miR-133a and miR-133b, whereas transfection of miR-133a or miR-133b precursors inhibited PKM2 expression in tongue squamous cell carcinoma cells [35]. The significance of this mutual antagonism between miR-133a/b and PKM2 has not been determined.

Recent studies revealed the molecular mechanism underlying PKM2 mRNA splicing. Heterogeneous nuclear ribonucleoproteins (hnRNP) I, A1, and A2 bind to RNA sequences encoded by exon 9 and inhibit PKM1-specific mRNA splicing [36, 37]. The c-Myc oncoprotein regulates transcription of hnRNPI, hnRNPA1 and hnRNPA2, resulting in preferential PKM2 isoform expression in cancer cells overexpressing c-Myc [36]. Mammalian target of rapamycin (mTOR), a serine/threonine protein kinase that regulates cell growth, cell survival, and protein synthesis, also stimulates PKM2 expression through activation of HIF-1 and c-Myc [38]. Thus, activation of transcription factors and kinases, and downregulation of microRNAs results in high expression of PKM2 in cancer cells. However, analysis of human tumor and non-tumor tissues from kidney, liver, lung, and thyroid using an absolute quantification approach by mass spectrometry revealed that PKM2 protein expression is predominant in both human tumor tissues and tissue-matched normal controls, suggesting that no switch from PKM1 to PKM2 is required for tumor development [39]. These findings challenge the conclusion, which was based on the analysis of cancer cell lines, that PKM2 overexpression is a hallmark of cancer cells [3]. The proteomic study found that total PKM expression (PKM1 + PKM2) is increased 3-fold in tumor tissue compared to normal tissue [39]. HIF-1α is overexpressed in solid tumors due to intratumoral hypoxia, genetic alterations, or both [12, 18, 22, 23], and thus may be a predominant regulator that contributes to elevated levels of PKM2 in human tumor tissues.

## PKM2 AND THE WARBURG EFFECT IN CANCER CELLS

Christofk *et al*. demonstrated that PKM2 expression was associated with increased glucose uptake and lactate production, but decreased O_2_ consumption in cancer cells [3]. Genetic manipulation of cancer cells that switched them from PKM2 to PKM1 expression reversed the Warburg effect, suggesting that high expression of PKM2 is required for aerobic glycolysis in cancer cells. Moreover, expression of PKM2, but not PKM1, induced tumor xenograft growth in nude mice [3]. The binding of tyrosine-phosphorylated peptides to PKM2 at lysine-433 was found to inhibit PKM2 enzymatic activity through release of the allosteric activator fructose-1,6-bisphosphate (FBP) and to promote cell growth and glycolytic metabolism in cancer cells [40]. Tyrosine kinases play critical roles in cell growth, cell metabolism, and angiogenesis in cancer [41, 42]. Hitosugi *et al*. reported that fibroblast growth factor receptor type 1 (FGFR1) phosphorylated PKM2 at tyrosine residues-83, -105, -148, -175, -370, and -390 in murine Ba/F3 hematopoietic cells [43]. Phosphorylation of PKM2 at tyrosine-105 induced FBP release from active tetrameric PKM2, promoted formation of less active dimeric PKM2, and subsequently decreased PKM2 enzymatic activity. In contrast, the phosphorylation of PKM2 at other tyrosine residues caused by FGFR1 failed to regulate PKM2 activity. However, it remained unclear how alterations in PKM2 activity could determine whether the product of the PKM2 reaction, pyruvate, was converted to lactate or to AcCoA (Figure 1).

**Figure 2:**
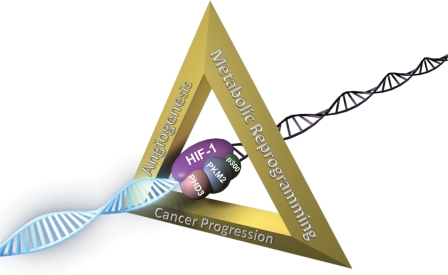
PKM2 contributes to metabolic reprogramming and cancer progression by serving as a PHD3-dependent coactivator for HIF-1 Prolyl hydroxylation of PKM2 by PHD3 promotes the interaction of PKM2 with HIF-1α, thereby stabilizing HIF-1 binding to the HRE of target genes, recruitment of coactivator p300, histone acetylation, and subsequent transcription of HIF-1 target genes, which encode proteins that are involved in metabolic reprogramming, angiogenesis, and many other critical aspects of cancer progression.

We recently delineated a molecular mechanism by which PKM2 mediates the Warburg effect in cancer cells [9]. PKM2 was found to interact with HIF-1α in the nucleus and to function as a transcriptional coactivator in HeLa cervical carcinoma and Hep3B hepatoblastoma cells. PKM2 increased HIF-1 binding to HREs at target genes, recruitment of coactivator p300, histone acetylation, and subsequent transactivation of HIF-1 target genes including *SLC2A1* (which encodes glucose transporter 1), *LDHA*, and *PDK1* in HeLa and Hep3B cells. PKM2-stimulated expression of HIF-1 target genes promotes the shift from oxidative phosphorylation to glycolytic metabolism. PKM2 also binds to HIF-2α and promotes HIF-2-mediated transactivation in cancer cells [9]. In addition to its effects on genes encoding metabolic enzymes, PKM2 stimulates HIF-1- and HIF-2-mediated expression of the *VEGF* gene (which encodes vascular endothelial growth factor), thereby promoting angiogenesis. Thus, PKM2 may play a far broader role in promoting cancer progression than was previously appreciated (Figure 2).

The enzymatic activity of PKM2 is not required for HIF-1 transactivation [9]. Interestingly, PKM2 is prolyl hydroxylated in the PKM2-specific domain encoded by exon 10, and prolyl hydroxylation of PKM2 is required for HIF-1-mediated transactivation in cancer cells. PHD3 catalyzes hydroxylation of PKM2 and PHD3 knockdown reduced expression of the HIF-1 target genes *SLC2A1*, *LDHA*, and *PDK1*, and reversed the Warburg effect in VHL-null RCC4 renal carcinoma cells [9]. PKM2, but not PKM1, is prolyl hydroxylated and PKM2, but not PKM1, interacts with HIF-1α, thus providing a molecular basis for the selective effect of PKM2 on HIF-1-mediated transactivation and the Warburg effect in cancer cells [9].

**Figure 3:**
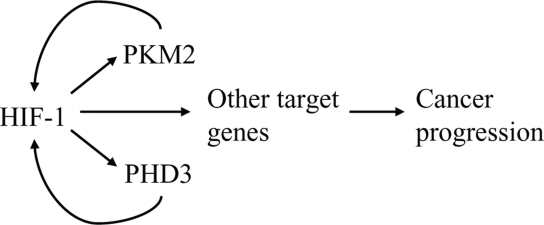
PKM2 and PHD3 are components of a positive feedback loop that amplifies HIF-1 transcriptional activity HIF-1 activates transcription of genes encoding PKM2 and PHD3, which interact with HIF-1α to stimulate transactivation of HIF-1 target genes that promote cancer progression.

Recently, the interaction of PHD3 with PKM2 was reported to increase the formation of dimeric PKM2, which has decreasd activity compared to the tetrameric form of the enzyme [44]. Chen *et al*. concluded that the hydroxylase activity of PHD3 was not required for its effect on PKM2 oligomerization/enzyme activity because mutant PHD3 (R205K) behaved similarly to wild-type PHD3. However, we found that mutation of arginine-205 alone was not sufficient to inactivate the hydroxylase activity of PHD3 (W.L. and G.L.S., unpublished) and thus it would be interesting to repeat these experiments using the PHD3 (H135A/D137A) mutant that lacks hydroxylase activity [9]. PHD3 is encoded by a HIF-1 target gene and increased PHD3 mRNA and protein expression is induced by HIF-1 under hypoxic conditions [45], which compensates for the reduced hydroxylase activity [9]. Thus, PHD3 and PKM2 exert a positive feedback loop in cancer cells that amplifies HIF-1 activity, which may play a major role in driving metabolic reprogramming, angiogenesis, and other critical aspects of cancer progression (Figure 3).

## UNANSWERED QUESTIONS, FUTURE DIRECTIONS

Does PKM2 have other functions in the nucleus that promote cancer progression? PKM2 also binds to the transcription factor Oct-4 and enhances Oct-4-dependent gene transcription [46]. Oct-4 is a key mediator of pluripotency in embryonic stem cells [47] and induced pluripotent stem cells [48]. Oct-4 is also expressed in human breast cancer stem cells [49]. Hoshino *et al*. also found that nuclear translocation of PKM2 is induced by interleukin-3 and stimulates cell proliferation [50], although the nuclear target of PKM2 was not identified. It is likely that the stimulation of cell proliferation by PKM2 is independent of its regulation of HIF-1α transactivation.

Post-translational modification by prolyl hydroxylation and tyrosine phosphorylation regulate PKM2 activity as a transcriptional coactivator and glycolytic enzyme, respectively. PKM2 is also subjected to sumoylation [51] and lysine acetylation [52] and further studies are required to determine whether these post-translational modifications also regulate the role of PKM2 as a HIF-1 coactivator.

Several compounds have been shown to inhibit PKM2 enzymatic activity [53, 54]. However, those inhibitors may not suppress HIF-1 transactivation in cancer cells because the enzymatic activity of PKM2 is not required for its coactivator functions [9]. Combination therapy with HIF inhibitors [12, 18, 19] may prove to be more efficacious.
